# A Perspective on How User-Centered Design Could Improve the Impact of Self-Applied Psychological Interventions in Low- or Middle-Income Countries in Latin America

**DOI:** 10.3389/fdgth.2022.866155

**Published:** 2022-06-02

**Authors:** Alejandro Dominguez-Rodriguez, Anabel De La Rosa-Gómez

**Affiliations:** ^1^Health Sciences Area, Valencian International University, Valencia, Spain; ^2^Faculty of Higher Studies Iztacala, National Autonomous University of Mexico, Mexico City, Mexico

**Keywords:** user center design (UCD), Latin America, online intervention, double diamond design process, mental disorders

## Abstract

Global technological progress has generated alternatives for psychological assistance, both for the evaluation and for the treatment of different emotional disorders. Evidence suggests that Internet-based treatments are effective for the treatment of anxiety and depression disorders. However, in Latin America online treatments are still scarce compared to developed countries and have similar problems as developed countries, such as high dropout rate. One possible solution to help decrease the dropout rate is to design and develop online interventions based on the needs and characteristics of the users. The user-centered design (UCD) is a fundamental concept to develop successful online interventions. The objective of this article is to provide a perspective overview on how UCD could improve the impact of self-applied psychological interventions in low- or middle-income countries in Latin America; however this proposal can also be applied in low- and middle-income countries in other regions of the world. The literature on UCD has demonstrated its efficacy when properly applied in online interventions; however, it is not common to see how this methodology has been applied in research in online interventions, and regarding Latin America, this is even more scarce with a very limited number of articles implementing the principles of UCD.

## Introduction

Before the Coronavirus SARS-CoV-2 (COVID-19) pandemic, the prevalence of anxiety and depressive disorders worldwide were already a matter of concern for international governments. According to the WHO, an approximate estimate of 322 million people suffered from depression and 264 million from anxiety disorders, with an increase of 18.4 and 14.9% respectively from 2005 to 2015 (1). In the same line, in the study of Santomauro et al. (2), published in The Lancet with data of 204 countries and territories during the pandemic of COVID-19, the authors indicated that there was an increase of 53.2 million more cases of major depression, and additionally, 76.2 million cases of anxiety disorders. Regarding Latin America, before the pandemic the mental disorders were perceived as common (3), and during the pandemic the available scientific literature indicates that the population of Latin American countries exhibited an increase in symptoms of anxiety (4), depression (5), post-traumatic stress disorder (6), sleep problems (7), and alcohol consumption (8), among other problems.

However, a vast majority of individuals in low- and middle-income countries do not receive psychological treatment for mental health disorders, mostly because it is not perceived as necessary. This could be related to a lack of health literacy, and mostly due to lack of infrastructure available to offer psychological treatment to the population (9). According to the Pan American Health Organization (PAHO), the public expense on mental health in the region of the Americas is barely 2% and it is mostly focused on psychiatric hospitals (10). Also, it has been observed that most of the mental health research is conducted in high-income countries, producing with this an imbalance where population in low and middle-income countries do not receive a proportional attention to their mental health needs (11). For example, in the scoping review of Jimenez-Molina et al. (3), the authors highlight the huge gap in the treatment of mental disorders in Latin America, and that this gap is bigger among socially disadvantaged groups, when the authors analyzed 22 studies on online interventions conducted in Latin America, where it was observed that the results were promising, however, higher quality studies are needed, especially Randomized Controlled Trials (RCT's), since of those 22 studies analyzed, only 4 were RCTs.

## E-Mental Health: Internet-Based Self-Applied Interventions

Global technological progress has generated alternatives for psychological assistance, both for the evaluation and for the treatment of different emotional disorders. Evidence suggests that Internet-based treatments are effective for the treatment of anxiety and depression disorders (12–14). Likewise, the meta-analysis data reveal that these interventions are as effective as face-to-face treatments (15, 16). Thus, on the one hand, there is evidence of the benefits shown by the interventions provided by the Internet and mobile applications by allowing: 1) accessibility: easy access at any time and from anywhere through the Internet, 2) flexibility: the intervention is adapted to the rhythm of the participant, and the consultation of resources and materials can be asynchronous, 3) personalization: the intervention can be adapted to the specific needs of the user, 4) availability of treatment: it allows to bring psychological care to people who need it regardless of distance, which constitutes an alternative when it is not possible to access face-to-face mental health services. In addition, 5) scalability since it can increase coverage and improve care as technology advances (17, 18). This means enhancing the scope and impact of psychological treatment programs for mental health problems (19). Due to these findings, online interventions are very accessible and can be used on a large scale, but they need to be further developed and implemented, especially focusing on the target population or users.

## Evidence-Based Practice: Relevance of User Opinion, Perspective, and Context for the Efficacy of Interventions

From the framework of Evidence-Based Psychotherapy, on the one hand, it is recommended to rigorously analyze the available scientific evidence to measure the efficacy of an intervention, that is, the therapeutic changes produced in controlled clinical trials. On the other hand, to measure effectiveness, it is suggested to investigate the generalization of the results of the intervention to the particular and cultural context of the target population; this is known as Evidence-Based Practice (EBP) and involves the integration of research knowledge with the clinical experience of the therapist and the characteristics, opinions, preferences, interests, values, context, and culture of the patient/user (20). Thus, it is incorporated as a priority to consider the opinions and perspectives of the patients/users in their own intervention as possible moderators and mediators of therapeutic adherence, improvement of patient engagement/commitment, treatment outcomes and reduction of therapeutic abandonment (21, 22). It has been reported that effective cultural adaptations considers age, disabilities, religion, ethnicity, sexual orientation, gender identity, socioeconomic status, indigenous group membership, and nationality. Also, patients' activity preferences include types of homework assignment, completion of worksheets, etc., delivery of treatment, patients' preferences language, and incorporation of cultural explanations of the specific disorder or treatment, and so on (20–23).

Regarding the efficacy of technological-based psychological interventions, specifically self-applied interventions, they have a series of challenges that involve the interaction of a great variety of factors at different systemic levels (24). However, the design features and specific functions of Internet-based intervention programs often receive less attention than the contents, and therefore do not contribute to meeting users' needs. Thus, key design problems could affect the generalizability of the results of effective clinical interventions at the time of their implementation in the community context (25, 26). Challenges include flexibility, complexity, and effectiveness of interventions, as well as studying the one-way relationship between program development and implementation (27).

## User-Centered Design in the Development of Psychological Interventions

Even though online interventions have several benefits, one of their main problems is the high drop-out rate, with percentages ranging from 1 to 50% for anxiety and depression treatments (24), and 43% for treatments for chronic diseases (28). In general, observational studies have higher dropout rates (49%, 95%), than RCTs (in more controlled scenarios) with 40% abandonment (29). Also, there is another issue related to the definitions of adherence not being well suited to the characteristics of e-interventions (28). Christensen et al., differentiate *adherence* as the extent to which individuals experience the content of internet intervention, and *dropout* describes an individual who fails to complete the research trial protocol associated with an internet intervention, and thus does not complete the trial assessments (28).

One possible solution to help decrease the drop-out rate and to increase the adherence is to design and develop online interventions based on the needs and characteristics of the user. Hence, the user centered design (UCD) is a fundamental concept to develop successful online interventions and decrease the drop-out rates. According to Abras et al. (30), “UCD is a broad term to describe design processes in which end-users influence how a design takes shape. It is both a broad philosophy and a variety of methods. There is a spectrum of ways in which users are involved in UCD, but the important concept is that users are involved one way or another” (30) (p1). In 2010 the standard ISO 9241-210 enriched the definition of UCD, referring to the UCD approach as Human-Centered Design (HCD) (31). Therefore, the ISO 9241-210 standard defines human-centered design as “an approach to systems design and development that aims to make interactive systems more usable by focusing on the use of the system and applying human factors/ergonomics and usability knowledge and techniques.” (31) (p2). The term UCD was coined by Donald Norman in the 1980s (30). In his book, The Design of Everyday Things, revised and expanded edition, Norman indicates that there are 4 activities in HCD: 1) Observation, 2) Idea Generation, 3) Prototyping, 4) Testing (32). An excellent example of how to implement the HCD is the study of Harte et al. (31), where the authors carried out and evaluated with a sample of older adults a methodology of 3 steps that followed the flow of ISO 9241-210, which included: 1) Established context of Use and User Requirements, 2) Expert Inspections and Walkthroughs and 3) Usability Testing with End users.

## Double Diamond Process in User-Centered Design

**Figure 1:**
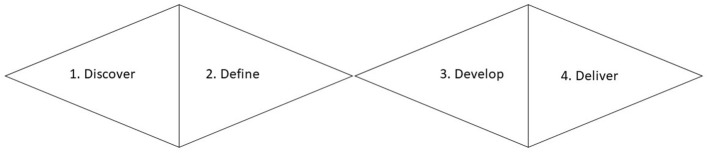
Double diamond design process.

When planning the design process, it is fundamental to talk about one of the most used design processes, the Double Diamond (Figure 1).

The double diamond design process illustrates how the design process goes from thinking and possibilities abroad as possible to situations that are reduced and are specially focused on different objectives (33).

The double diamond phases are detailed in: 1) Discover, where the start of a project is a period of discovery, gathering inspiration and insights, identifying user needs and developing initial ideas, 2) Define, the second quarter represents the definition phase, in which designers try to make sense of all the possibilities identified in the Discover phase, 3) Develop, the third quarter marks a period of development where solutions are created, prototyped, tested, and iterated. This process of trial and error helps designers to improve and refine their ideas and 4) Deliver, the final quarter of the double diamond model is the Deliver phase, where the resulting product or service is finalized and launched. The key activities and objectives during this stage are the following: final testing, approval and launch, targets, evaluation, and feedback loops (33) (p7). There are excellent examples for developing online programs to deliver telehealth and that apply the double diamond model such as Banbury et al. (34), where the researchers aimed to develop a telehealth peer support program for isolated dementia caregivers. Another example is the study of Hardy et al. (35), where the researchers implemented the double diamond method to improve psychological therapies for psychosis. While there are benefits to designing and delivering interventions online following the double diamond process, it is still not the norm.

Regarding Latin America, the UCD is not commonly mentioned or indicated in online interventions, with only few exceptions such as Antelo et al. (36), where the authors presented a Counseling Mobile App designed on the UCD approach to women in low- and middle-income setting in Argentina aimed at the reduction of the psychosocial impact of the human papilloma virus. In Mexico, Dominguez-Rodriguez et al., (37) designed, developed, and implemented a self-applied intervention based on user experience (UX) principles in order to decrease the risk of developing complicated grief disorder, increase the life and sleep quality, and reduce symptoms of anxiety and depression. Also in Mexico, Pérez-Bautista et al. (38), designed and evaluated an online intervention for young Mexican deaf for transfer of sexual education using a UCD design and involving objective participants (deaf youngs) on the design process. In Colombia, Ospina-Pinillos et al. (39), adapted the Web-based Mental Health eClinic (MHeC) that was adapted for the Spanish population living in Australia (MHec-S) (40) and coming from the original version of the MHeC that was designed in Australia (41). The researchers adapted this tool to the Colombian population using participatory design methodologies. Also, there are some studies where online interventions have been delivered in Latin American populations, specifically in Chile and Colombia that did not successfully engage the users (42). The authors concluded that it is necessary to improve the intervention results by increasing its levels of customization and by developing strategies to achieve better adherence. Therefore, the importance of considering the UCD at the moment of proposing, designing, developing, and delivering online mental health interventions is highlighted. Further online interventions applying UCD principles in Latin America are scarce.

There are several developments of digital mental health tools that are user-centered for several purposes. For example, the low-intensity intervention for Syrian refugees (43), part of the EU Horizon 2020 STRENGTHS (Syrian REfuGees MeNTal HealTH Care Systems) program (44), in which participants reacted positively to the prototype presented, making emphasis on the potential health impact of the intervention, the flexibility and customizability of it, the easiness to learn how to use it, and the aesthetic components. In other contexts, regarding online interventions but focused on adherence for medical treatments such as hydroxyurea for sickle cell disease, where the participants report poor adherence, Alberts et al. (45) used a user-centered approach to investigate the reasons for poor adherence and based on the health belief model analyzing the benefits, barriers, the researchers identified the possible reasons for poor adherence and adapted an mHealth solution to overcome these barriers through an App call *InCharge Health*. The study of UCD and attrition reduction is also being applied in other contexts such as web-Based Psychological Intervention for Patients with Myocardial Infarction with Non-obstructive Coronary Arteries, where the researchers designed a UCD web-based psychological 9-step program focusing on stress, worry, and valued action for persons with such health conditions, and among the several objectives of this tool was also to reduce the attrition of the participants on this intervention (46).

On the other hand, there are currently studies trying to implement interventions based on HCD in low-resource communities (47); however, the results are still not available.

## Discussion

The objective of this article was to provide a perspective overview on how user-centered design could improve the impact of self-applied psychological interventions in low- or middle-income countries in Latin America, however this proposal can also be applied in low- and middle-income countries in other regions of the world.

The literature is still being gathered, but there are excellent examples of how UCD can be implemented in online interventions (30, 31, 34–43). For countries in Latin America where the budget is limited for mental health interventions, we recommend that the researchers focus on learning and applying the UCD principles that were briefly presented in this article. Due to the COVID-19 pandemic, there is currently an urgent need for online mental health interventions, and we predict that other problematics such as economic, political, and/or climate change crisis will require the support of evidence-based psychological treatments that can widely reach the general population, and that will be perceived by the users as easy to use and enjoyable to return to use them and even to recommend them to other persons.

This manuscript has certain limitations that should be considered. For example, the literature on UCD has demonstrated its efficacy when properly applied in online interventions, however it is not common to see how this methodology has been applied in research in online interventions, and regarding Latin America this is even more scarce with a very limited number of articles implementing the principles of UCD and to the best of the knowledge of the authors, none clearly stating to be implementing the double diamond model. More research needs to be conducted to provide evidence-based treatments to the population of Latin America and the Caribbean.

In this regard, this article shows a perspective based on the review of the existing literature, it is suggested for future studies to carry out a meta-analysis on the effectiveness of the interventions and their relationship with the design centered on the user experience in the context of Latin America.

## Data Availability Statement

The original contributions presented in the study are included in the article/supplementary material, further inquiries can be directed to the corresponding author/s.

## Author Contributions

All authors listed have made a substantial, direct, and intellectual contribution to the work and approved it for publication.

## Conflict of Interest

The authors declare that the research was conducted in the absence of any commercial or financial relationships that could be construed as a potential conflict of interest.

## Publisher's Note

All claims expressed in this article are solely those of the authors and do not necessarily represent those of their affiliated organizations, or those of the publisher, the editors and the reviewers. Any product that may be evaluated in this article, or claim that may be made by its manufacturer, is not guaranteed or endorsed by the publisher.

## Abbreviations

EBPs: Evidence-based practice
HCD: Human-Centered Design
ISO: International Standards Organization
PAHO: Pan American Health Organization
RCTs: Randomized Controlled Trials
COVID-19: SARS-CoV-2
UCD: User-centered design
WHO: World Health Organization.
